# Modern Approaches to Quality Assurance of Drug Formulations

**DOI:** 10.1155/2015/126478

**Published:** 2015-05-20

**Authors:** Josef Jampilek, Patrick J. Crowley, Mark Olsen, Kin Tam

**Affiliations:** ^1^Department of Chemical Drugs, Faculty of Pharmacy, University of Veterinary and Pharmaceutical Sciences Brno, Palackeho 1/3, 612 42 Brno, Czech Republic; ^2^Callum Consultancy LLC, 571 Berwyn Baptist Road, Devon, PA 19333, USA; ^3^Department of Pharmaceutical Sciences, College of Pharmacy, Midwestern University, Glendale Hall 236-18, 19555 N. 59th Avenue, Glendale, AZ 85308, USA; ^4^Faculty of Health Sciences, University of Macau, Avenida da Universidade, Taipa, Macau

Developments in pharmaceutical sciences have provided sophisticated oral dosage forms to modify drug action by delaying its onset or by slowing or otherwise changing the rate of delivery to reduce side effects or sustain drug action by maintaining constant plasma levels. Delivery of active pharmaceutical ingredients with suboptimal physicochemical properties to the targeted sites could be achieved using recently developed new formulation technologies. More novel modes of delivery have also gained prominence. These may provide (e.g.) transdermal delivery or time-based (chronotherapeutic) release or may target specific organs, tissues, or cellular structures. Numbers of biopharmaceutical products have also increased and will probably continue to do so. These too may benefit from formulating for controlled or site-specific delivery.

Such novel systems, therapeutic agents, and evolving paradigms place increasing demands for assuring and controlling product quality. At the same time they provide opportunities for developing and putting into practice new ideas and technologies to assure quality and performance. Well-designed dosage forms and processes, along with process analytical technologies, can provide insights and online monitoring systems that could largely replace end product testing. Manufacture, testing, and batch approval times could be greatly reduced.

The same principles apply to manufacture and quality assurance of drug-device systems, providing assurance of performance related to amount, rate, and possibly time of drug delivery.

In this special issue readers can find examples of preparation and characterization of modern sophisticated drug delivery systems such as oral mucoadhesive films, wound dressing films, solid dispersions, or liquisolid systems for enhancing dissolution rate and improving* in vivo* bioavailability of poorly soluble drugs. Unique site-specific or controlled release antimicrobial systems based on carmellose cross-linked by copper ions are also described. A paper also focuses on the preparation and characterization of silica-based nanocarrier loaded nootropics, which are designed to enhance the permeation of the drugs from the circulatory system through the blood-brain barrier. Other papers deal with the assurance of medical device quality with quality management systems, quality by design, innovative analytical techniques, and their applications in process monitoring, analysis and control, determination of critical quality attributes, and* in vitro in vivo* correlations based on multicompartmental dissolution data.

*Josef  Jampilek* *Patrick  J.  Crowley* *Mark  Olsen* *Kin  Tam*

